# Discovering the Deregulated Molecular Functions Involved in Malignant Transformation of Endometriosis to Endometriosis-Associated Ovarian Carcinoma Using a Data-Driven, Function-Based Analysis

**DOI:** 10.3390/ijms18112345

**Published:** 2017-11-06

**Authors:** Chia-Ming Chang, Yi-Ping Yang, Jen-Hua Chuang, Chi-Mu Chuang, Tzu-Wei Lin, Peng-Hui Wang, Mu-Hsien Yu, Cheng-Chang Chang

**Affiliations:** 1 School of Medicine, National Yang-Ming University, Taipei 112, Taiwan; cm_chang@vghtpe.gov.tw (C.-M.C.); molly0103@gmail.com (Y.-P.Y.); chuangjenhua5@gmail.com (J.-H.C.); cmjuang@gmail.com (C.-M.C); backyard0826@gmail.com (T.-W.L.); phwang@vghtpe.gov.tw (P.-H.W.); 2 Department of Obstetrics and Gynecology, Taipei Veterans General Hospital, Taipei 112, Taiwan; 3 Department of Medical Research, Taipei Veterans General Hospital, Taipei 112, Taiwan; 4 Department of Medical Research, China Medical University Hospital, Taichung 404, Taiwan; 5 Department of Obstetrics and Gynecology, Tri-Service General Hospital, National Defense Medical Center, Taipei 114, Taiwan; hsienhui@ms15.hinet.net

**Keywords:** endometriosis, ovarian carcinoma, function-based, data-driven analysis, microarray gene expression datasets, Gene Ontology

## Abstract

The clinical characteristics of clear cell carcinoma (CCC) and endometrioid carcinoma EC) are concomitant with endometriosis (ES), which leads to the postulation of malignant transformation of ES to endometriosis-associated ovarian carcinoma (EAOC). Different deregulated functional areas were proposed accounting for the pathogenesis of EAOC transformation, and there is still a lack of a data-driven analysis with the accumulated experimental data in publicly-available databases to incorporate the deregulated functions involved in the malignant transformation of EOAC. We used the microarray gene expression datasets of ES, CCC and EC downloaded from the National Center for Biotechnology Information Gene Expression Omnibus (NCBI GEO) database. Then, we investigated the pathogenesis of EAOC by a data-driven, function-based analytic model with the quantified molecular functions defined by 1454 Gene Ontology (GO) term gene sets. This model converts the gene expression profiles to the functionome consisting of 1454 quantified GO functions, and then, the key functions involving the malignant transformation of EOAC can be extracted by a series of filters. Our results demonstrate that the deregulated oxidoreductase activity, metabolism, hormone activity, inflammatory response, innate immune response and cell-cell signaling play the key roles in the malignant transformation of EAOC. These results provide the evidence supporting the specific molecular pathways involved in the malignant transformation of EAOC.

## 1. Introduction

Endometriosis**-**associated ovarian carcinoma (EAOC), which includes ovarian clear cell carcinoma (CCC) and endometrioid carcinoma (EC) [1,2], are relatively rare subtypes of ovarian cancer. Compared to other ovarian cancers, chemoresistance and worse prognosis are unsolved issues in CCC [3,4]. Another clinical characteristic of EAOC is the frequent occurrence of ovarian endometrioma. Endometriosis is related to a 2–3-fold increase of EOC incidence and is also found in 15–20% of CCC and EC [5,6,7]. Previous studies also indicated that approximately 1% of endometrioma give rise to ovarian cancer [1,2,8]. Furthermore, the presence of atypical endometriosis in 36% of CCC and 23% of EC [9], regarded as large nuclei and increased nuclear-cytoplasmic ratio, occurs in 8% of endometrioses [10]. Most atypical endometriosis revealed a direct continuity with CCC or EC [11] and reflected the precancerous lesions of CCC or EC. These clinical observations demonstrated the close association between endometriosis and CCC/EC. However, the results of the investigations for the malignant transformation of EAOC still vary and are inconclusive.

Based on the clinical findings and published experimental data, there is still a lack of analysis to explore the functions, as well as their relationship, systemically by incorporating the abundant DNA microarray datasets with the publicly-available databases. Herein, we utilized a function-based model established with the whole human functions, i.e., functionome, to investigate the functional aberrations of complex diseases. We downloaded 1454 Gene Ontology (GO) gene sets with defined functions from the Molecular Signatures Database (MSigDB) [12]. Then, we utilized these 1454 GO term gene sets to measure the human genome-wide functionome. Each function is quantified by measuring the gene expression regularity of the genes in that gene set and is defined as the gene set regularity (GSR) index. Using the GSR model, we have successfully quantified the functions of ovarian serous carcinoma (SC) at different stages during disease progression and demonstrated that the functions deteriorate in an almost linearly, stepwise fashion from the International Federation of Gynecology and Obstetrics (FIGO) Stage I–Stage IV [13]. In the second study, we demonstrated that the functional regulation patterns of CCC, EC and mucinous carcinoma are different from that of serous carcinoma [14]. This finding is compatible with the Type I and II classifications proposed by the widely-accepted dualistic model of ovarian carcinogenesis [15].

In this study, we further investigate the pathogenesis of EAOC by analyzing the genomic-wide functions involved in the malignant transformation with this data-driven analysis based on the functionomes of ES, CCC and EC. The informativeness of the functionomes were first analyzed by the accuracies of recognition, classification and prediction with machine learning; then, the crucial deregulated functions involved in the pathogenesis of EAOC were extracted by a workflow consisting of biostatistical methods, exploratory factor analysis (EFA) and ranking analysis through a series of logistic filters.

## 2. Results

### 2.1. Workflow the Functional Regularity Model

**Figure 1:**
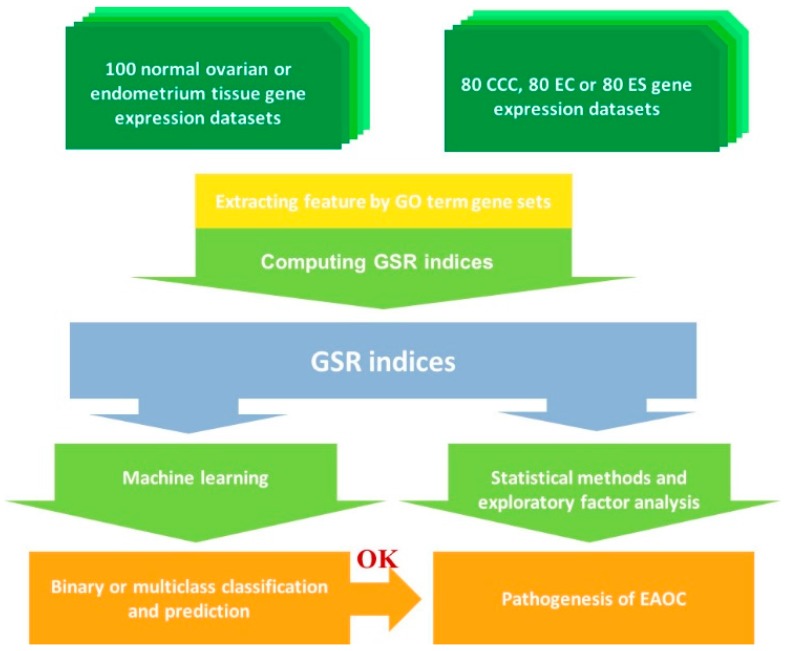
Workflow of the gene set regularity model. The gene set regularity (GSR) index was computed by converting the gene expression ordering of gene elements in a gene set through the Gene Ontology (GO) term or canonical pathway databases. The genome-wide informativeness of the GSR index was assessed by the accuracy of pattern recognition, classification and prediction by machine learning using binary or multiclass classifications. Functionome analyses were carried out to investigate the pathogenesis of endometriosis (ES), clear cell carcinoma (CCC), endometrioid carcinoma (EC) and endometriosis associated ovarian carcinoma (EAOC) by statistical methods, hierarchical clustering and exploratory factor analysis.

The workflow of the GSR model is displayed in Figure 1, and the detail of the algorithm is described in the Materials and Methods Section. This workflow consists of the following analytic procedures: (1) Computing the GSR indices: The extracted gene expression profiles are converted to the quantified 1454 GO term functions based on the gene expression orderings of the gene elements in each gene set. This quantified function, i.e., the GSR index, ranged from 0–1; one represents that the regularity in a gene set is not changed between the case and the most common gene expression orderings in the normal controls, while zero represents that the gene set regularity is in the most chaotic state. (2) Checking the functional regularity patterns and the informativeness of the genome-wide functionome: The informativeness of the functionome consisting of the 1454 GSR indices is evaluated with the accuracies of classification and prediction by machine learning. (3) Investigation of EAOC pathogenesis: In the final step, the key deregulated functions involved in the malignant transformation of ES to CCC or EC are extracted by a secession of analytic procedures and filters, including the exploratory factor analysis (EFA) and ranking analysis.

### 2.2. DNA Microarray Gene Expression Datasets and Gene Set Definition

The microarray gene expression profiles for ES, CCC, EC and the normal control samples were downloaded from the Gene Expression Omnibus (GEO) database, including 80 ES, 100 normal endometrium controls, 80 CCC, 80 EC and 100 normal ovarian tissue control samples (Table 1). These samples’ data were collected from 39 datasets containing seven different DNA microarray platforms without missing data. The detailed sample information, including the staging, DNA microarray platforms, dataset series and accession number are available in Table S1. The version of the GO term gene set definitions downloaded from the MSigDB was “c5.all.v5.1.symbols.gmt (2016)”, containing 1454 gene sets. Due to the different genes examined in different microarray platforms, a final 1453, 1447 and 1446 GO gene sets were utilized in the ES, CCC and EC groups, respectively. Finally, the 1446 common gene sets were utilized for the GSR model in this study.

### 2.3. The Most Significantly Deregulated GO Terms

The 1446 common GO terms among the three diseases were ranked by their *p*-values to show the top deregulated functions. Table 2 displays the 15 most deregulated GO terms for ES, CCC and EC; all were statistically significant. Among the top seven deregulated GO terms for ES, most of them were related to transport activity. The 10th most deregulated GO term was “MAPK kinase kinase kinase, activity” (GO:0004709). The MAPK pathway is a well-known pathway related to endometriosis and involves inflammatory processes [16]. The top three deregulated GO terms for CCC were “Rho guanyl nucleotide exchange factor activity”, “cofactor transport” and “inositol or phosphatidylinositol phosphatase activity”, which were the 12th, 11th and 13th deregulated GO terms for EC. In general, the deregulated GO terms between CCC and EC were quite similar and just different in the rankings. The full tables of the GO terms and the corresponding *p*-values are available in Table S2.

### 2.4. Means and Histograms of GSR Indices of the Three Diseases

**Figure 2:**
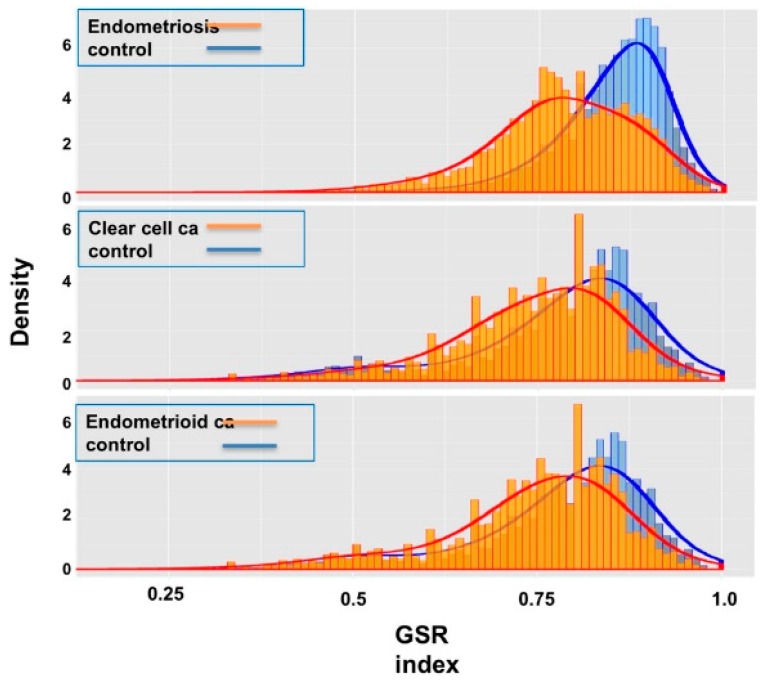
Histograms of the gene set regularity indices for the three diseases and control groups. The figures show the distributions of gene set regularity (GSR) indices from the three diseases (orange) and control groups (blue). The control group for CCC and EC is identical.

Table 2 displays the mean and standard deviation (SD) of the GSR indices for the three diseases and the normal tissue controls. The means of GSR indices for the three diseases are significantly lower than the controls, indicating that the functions are generally deregulated in ES, CCC or EC compared to the normal control group. Additionally, the means of the GSR indices of CCC and EC are lower than ES, revealing a worse functional regularity in CCC and EC. When displayed in the histograms (Figure 2), the distributions of the total GSR index levels between CCC and EC are quite similar, indicating the close relationship of functional regularity patterns between these two cancers; in contrast, the ES shows a different pattern of functional regularity from CCC and EC.

### 2.5. Close Relationship between CCC and EC

We utilized the set operations to find out the deregulated functions in common among ES, CCC and EC. The first 140, i.e., the top 10% of significantly deregulated GO terms were selected for the set operations. Notably, CCC and EC share as high as 60.71% of the deregulated functions (85/140), revealing the probably homogeneous etiology for the two cancers. ES shares 5.71% and 7.85% of the deregulated functions with CCC and EC, respectively. There are 2.85% (4/140) coexisting deregulated GO terms among the three diseases. The coexisting deregulated functions between ES with CCC/EC reveal the candidate deregulated functions’ response for the malignant transformation to CCC or EC.

**Figure 3:**
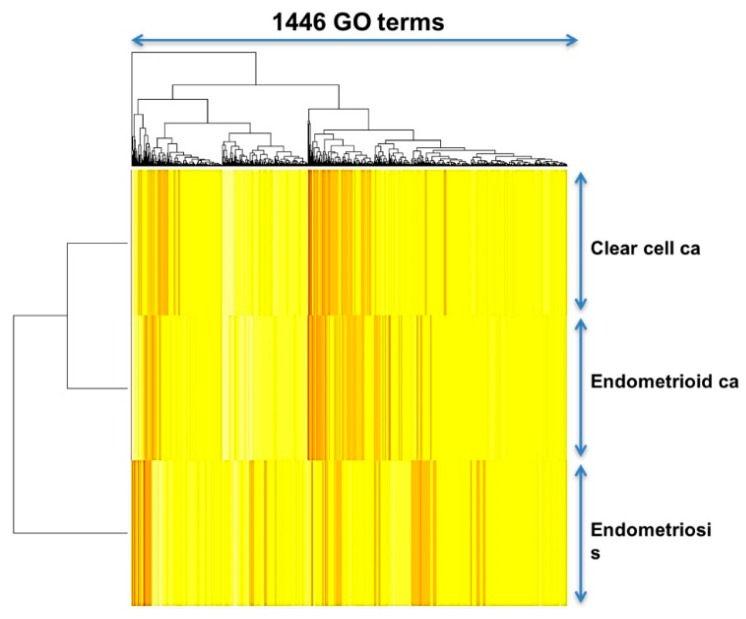
Heatmaps and dendrogram for the three diseases. The dendrogram (left side of the heatmap) shows the relationship of the three diseases. When displayed on the heatmap, each of the three diseases computed through the Gene Ontology (GO) term gene sets show a distinct pattern, however, the patterns are more similar between CCC and EC.

Unsupervised classification by the hierarchical clustering was utilized to uncover the relationship of the three diseases as the dendrogram shown on the left side of Figure 3, revealing an obviously close relationship between CC and EC. When displayed on the heatmap (Figure 3), CCC and EC also show similar patterns of functional regularity. These results indicate that most of the deregulated molecular functions and biological processes were similar and overlapping between CCC and EC.

### 2.6. High Classification and Prediction Accuracies for the Functional Regularity Patterns of the Three Diseases by Machine Learning

The functional regularity patterns of the three diseases were recognized by machine learning, and then, the genome-wide informativeness of the GSR indices was evaluated by the accuracies of classification and prediction. Supervised classification was performed by support vector machine (SVM), and the performance was assessed with the accuracies of the binary and multiclass classification for the matrices of the GSR index computed from the total samples computed through 1454 GO term gene sets. The performance was tested by five-fold cross-validation. The results show that the accuracies of binary classification (case vs. control) are up to 98.88% for the ES, 99.72% for the CCC and 98.61% for the EC groups. The areas under the curve (AUCs) range from 0.9834–0.9965 (Table 3). The accuracies of multiclass classification among the three diseases and the normal control group were 98.2%. The high accuracies indicate that the GSR indices can provide sufficient information for machine learning to recognize and undergo adequate recognition and classification. It also reveals that the functional regularity patterns are distinct and can be applied to the molecular classification among the gene expression profiles of ES, CCC and EC.

### 2.7. Existence of Commonly Deregulated Functions among ES, CCC and EC Discovered by Exploratory Factor Analysis and GO Tree Mapping

**Figure 4:**
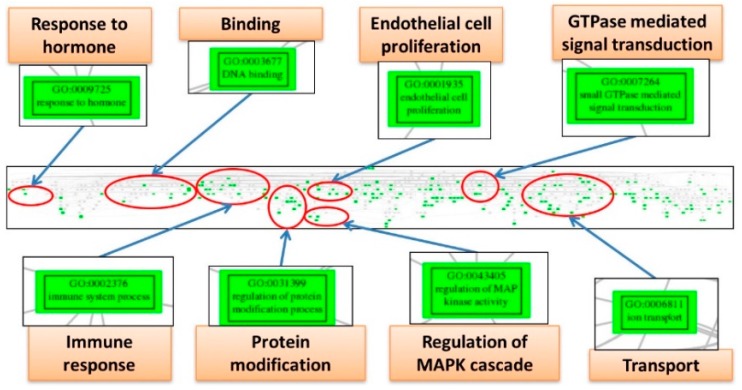
The Gene Ontology tree of deregulated functions from exploratory factor analysis for endometriosis. The figure displays the screenshot of the full Gene Ontology (GO) tree for endometriosis (ES) (middle panel). After mapping to the GO tree, the similar or related GO terms are clustered together. Each cluster is circled (red), and the important deregulated GO terms (green boxes) in the cluster are magnified to view the details. Each cluster is labeled by the common parental GO term (orange rectangle).

**Figure 5:**
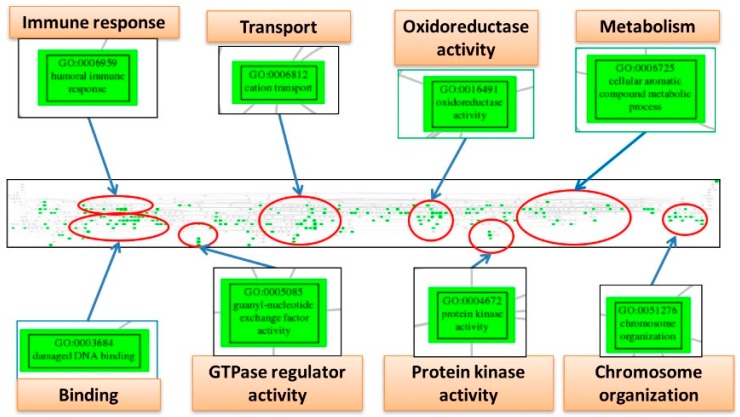
The GO tree of deregulated functions from exploratory factor analysis for clear cell carcinoma. This figure displays the screenshot of the full GO tree for ovarian clear cell carcinoma (CCC) (middle panel). After mapping to the GO tree, the similar or related GO terms are clustered together. Each cluster is circled (red), and the important deregulated GO terms (green boxes) in the cluster were magnified to view the details. Each cluster is labeled by the common parental GO term (orange rectangle).

**Figure 6:**
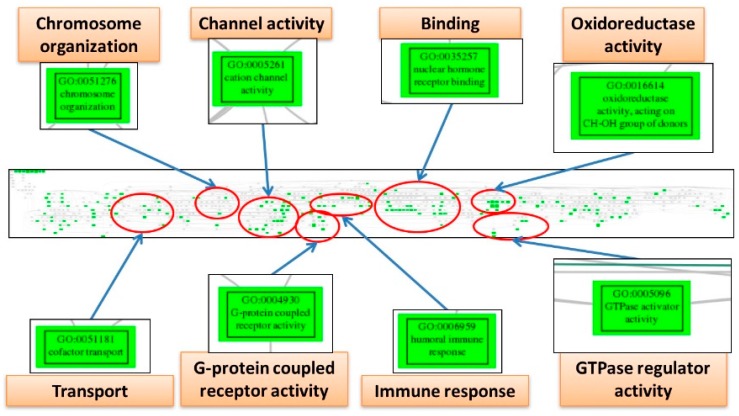
The GO tree of deregulated functions from exploratory factor analysis for endometrioid carcinoma. This figure displays the screenshot of the full Gene Ontology (GO) tree for ovarian endometrioid carcinoma (EC) (middle panel). After mapping to the GO tree, the similar or related GO terms are clustered together. Each cluster is circled (red), and the important deregulated GO terms (green boxes) in the cluster are magnified to view the details. Each cluster is labeled by the common parental GO term (orange rectangle).

The EFA can detect the underlying structure among numerous variables, so we utilized it to discover the core elements involving the pathogenesis network among the significantly deregulated GO terms for the three diseases. The numbers of factor, i.e., the group numbers of the interaction network, are 5, 5 and 3; each containing 391, 264 and 281 elements for ES, CCC and EC. To further concentrate the numerous GO terms and remove the redundant elements, we mapped them to the GO tree based on their parent-child hierarchy. The offspring GO terms on the GO tree were then clustered together so that the redundant GO terms were reduced, and the crucial deregulated functions involved in the pathogenesis of each disease could be summarized by tracing the common ancestral GO terms for each cluster; and the EAOC pathogenesis network viewed macroscopically. The pathogenesis network elements of ES can be summarized as the deregulated functions including “response to hormone”, “binding”, “endothelial cell proliferation”, “guanosine triphosphatase (GTPase)-mediated signal transduction”, “immune response”, “protein modification”, “regulation of MAPK cascade” and “transport” (Figure 4). The pathogenesis network elements of CCC can be summarized as the deregulated functions including “immune response”, “transport”, “oxidoreductase activity”, “metabolism”, “binding”, “GTPase regulator activity”, “protein kinase activity” and “chromosome organization” (Figure 5). The pathogenesis network elements of EC can be summarized as the deregulated functions including “chromosome organization”, “channel activity”, “binding”, “oxidoreductase activity”, “transport”, “G-protein coupled receptor activity”, “immune response” and “GTPase regulator activity” (Figure 6). Obviously, there are many deregulated functions coexisting between CCC and EC, such as “binding”, “immune response”, “oxidoreductase activity”, “chromosome organization”, “GTPase regulator activity” and “protein kinase activity”. The common deregulated functions among ES, CCC and EC are “immune response”, “GTPase activity” and “oxidoreductase activity”. These coexisting deregulated functions indicate the candidates involved in EAOC pathogenesis. The full table of these factors and the elements for ES, CCC and EC are available in Tables S3–S5. The full figures of the GO trees for the three diseases are available in Figures S1–S3.

### 2.8. Inflammation, Immune Response, Cell Division, Hormone Activity, Cell-Cell Signaling, Metabolism and Oxidoreductase Activity are the Core Functions Involved in the Malignant Transformation of EAOC

**Figure 7:**
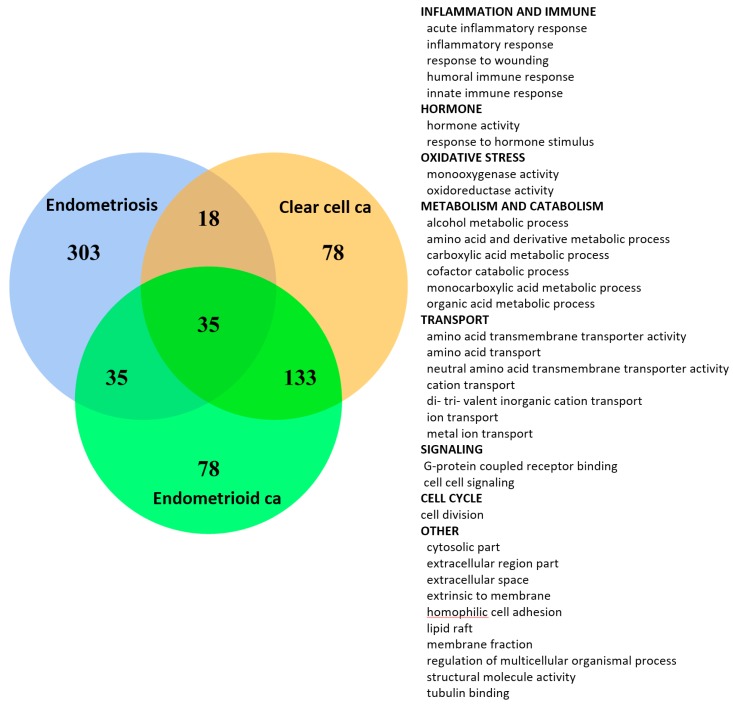
Venn diagram of the deregulated GO term elements from exploratory factor analysis for the three diseases. The figure shows the results of the three diseases with the total factor elements for each disease. Their relationship is displayed on the Venn diagram to show the gene set numbers of all possible logical relations among the three diseases. The 35 commonly-deregulated GO terms among ES, CCC and EC are listed on the right side of the figure.

**Figure 8:**
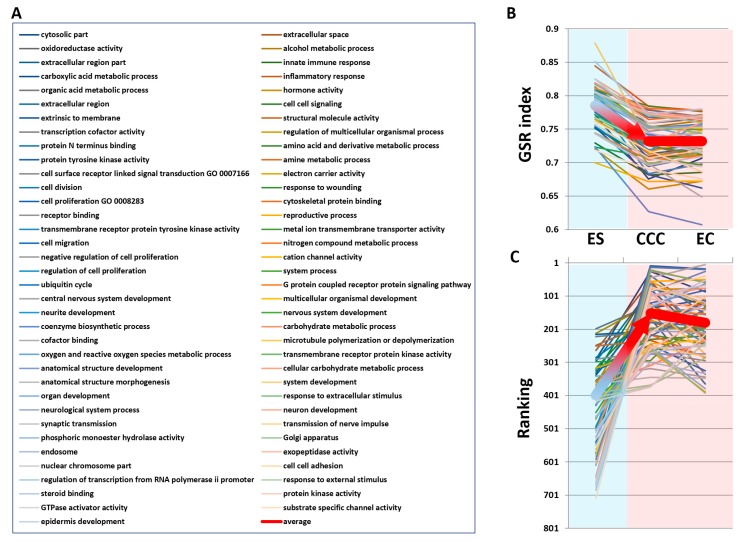
The 71 progressively-deregulated GO terms involving malignant transformation and their changes in GSR index and ranking from ES to CCC or EC. (**A**) The list of 71 progressively-deregulated GO terms whose GSR indices decreased and moved upward in rankings with the progression from ES to EAOC. (**B**) The GSR index levels of the 71 GO terms that decreased from ES to CCC or EC. (**C**) The ranking paths of the 71 GO terms changing from ES to CCC or EC. The coarse red line shows the paths for the average of the GSR indices (**B**) or rankings (**C**).

Based on the existence of common pathogenesis of the three diseases, we further looked for the detailed deregulated functions involved in the malignant transformation by selecting the coexisting deregulated functions from these EFA elements. We carried out the set operations to discover the coexisting deregulated functions among ES, CCC and EC EFA elements, and the results are displayed on the Venn diagram (Figure 7). CCC and EC share the most coexisting deregulated functions (35 + 133 = 168), indicating the similar pathogenesis between these two cancers. There are 35 commonly-deregulated functions among ES, CCC and EC, as shown in the right list in Figure 7. They could be summarized as the following functions, including “inflammation response”, “immune response”, “hormone”, “oxidative stress”, “metabolism”, “transport”, “signaling”, “cell cycle” and others. This result shows high consistency with our knowledge about the pathogenesis of EAOC. Then, we applied a second filter to extract the progressively deregulated functions for those significant deregulated GO terms whose GSR index levels were depressed and the rankings moved upward from ES to CCC or EC. The ranking paths of the selected functions from ES to CCC and EC are displayed on the line chart (Figure 8). There were 71 GO terms that met the selection criteria, as shown in the right panel of Figure 8. These GO terms represented the functions progressively deregulated, and their roles became more and more important as disease transition from ES to CCC or EC. These GO terms can be summarized as the following functions, including “immune response”, “inflammation response”, “hormone activity”, “cell signaling”, “transcription cofactor activity”, “binding”, “metabolism”, “cell division”, “development”, “oxidative stress”, “cell adhesion” and “GTPase activity”.

Finally, we summed up the principle deregulated functions from the 35 significant GO terms that were coexisting among the three diseases and the 71 GO terms whose GSR index levels decreased and moved upward in the rankings from ES to CCC or EC. This selection resulted in 17 GO terms, including “alcohol metabolic process”, “amino acid and derivative metabolic process”, “carboxylic acid metabolic process”, “cell-cell signaling”, “cell division”, “cytosolic part”, “extracellular region part”, “extracellular space”, “extrinsic to membrane”, “hormone activity”, “inflammatory response”, “innate immune response”, “organic acid metabolic process”, “oxidoreductase activity”, “regulation of multicellular organismal process”, “response to wounding” and “structural molecule activity”. With the results from the above function-based analyses through a succession of filters, the 17 core members of EAOC pathogenesis involved in the pathogenesis of EAOC can be summarized as the following functions, including “metabolism”, “cell division”, “cell-cell signaling”, “hormone activity”, “inflammatory response”, “innate immune response” and “oxidoreductase activity”.

To grossly view the deregulated functions involved in the pathogenesis of EAOC, as well as their interactions, we reconstructed the function network of CCC/EC by merging the CCC and EC datasets to re-compute the GSR indices to simulate the network of EAOC pathogenesis based on the mutual information. The largest subnetwork consists of 479 GO terms, and we display it with Cytoscape [17] as shown in Figure S4. The figure revealed complicated interactions among the deregulated functions (red circles) and the non-deregulated functions (green circles). As a complex disease, the interactions among these deregulated functions are complex and intensive, indicating that the etiology of EAOC cannot be explained merely by a single deregulated function.

## 3. Discussion

In order to understand the EAOC pathogenesis from a macroscopic view, we conducted this function-based, data-driven analysis to investigate complex diseases with the functionomes. The gene-gene interactions are taken into account during computation of the change of expression ordering of gene elements in a gene set. Additionally, this model converts gene expression profiles to gene expression orderings in the ordinal data. This data type will encounter less bias during cross-platform integration of gene expression datasets than the gene expression levels. These features make the GSR model feasible to integrate microarray gene expression datasets using different microarray platforms, to investigate the pathogenesis of EAOC through analyzing the genome-wide functionomes of different complex diseases and provides a more comprehensive and intuitive way to view the whole human functions. We demonstrated that the patterns of functional regulation of ES, CCC and EC could be accurately recognized and classified by unsupervised classification with hierarchical clustering and supervised classification by SVM. These findings demonstrate that the informativeness provided by the GSR indices is sufficient to make a clear distinction among the three diseases.

Our results show the inflammation and immune-related GO terms, including “MAP kinase kinase kinase activity” (GO:0008394, 10th deregulated GO term) and “activation of immune response” (GO:0002253, 14th deregulated GO term), significantly deregulated among ES, CCC and EC. However, the information of these significantly deregulated functions is not enough to reconstruct the underlying structure of ES pathogenesis. Thus, we utilized the EFA to discover the network of ES pathogenesis and further summarized the network elements by mapping them to the GO trees based on their GO hierarchies. These analyses reveal the crucial elements involving in the pathogenesis network of ES, such as “response to hormone”, “endothelial cell proliferation”, “inflammation response”, “immune response”, “regulation of MAPK cascade” and “oxidative stress”.

We also applied this workflow to investigate the pathogenesis of CCC and EC. The significantly deregulated GO terms and the results of EFA for the CCC and EC are quite similar, only differing in their positions in the functionome. Comparing the deregulated functions among the three diseases, the CCC and EC show similar function regularity patterns, revealing the possibility of homogeneous etiology between CCC and EC. Moreover, 60.7% of the top deregulated functions coexisted between CCC and EC, but only 2.85% deregulated functions coexisted among ES, CCC and EC, showing the possible deregulated functions involved in the pathogenesis of the malignant transformation of EAOC. We explored these coexisting deregulated functions from the most important network elements in ES, CCC and EC extracted from the EFA, including “inflammation response”, “immune response”, “hormone response” and “oxidative stress”. These results are consistent with the well-known aberrant functions or pathways in EAOC pathogenesis, indicating a high correlation between the coexisting deregulated functions and the malignant transformation.

We filtered the key members among the coexisting deregulated functions by selecting deregulated functions that were coexisting among the three diseases, that had functional regularity significantly depressed and for which the ranking in the functionome was upward-moving with disease transformation from ES to CCC or EC. This filter further extracted the crucial deregulated functions involved in the malignant transformation of EAOC, including metabolism, cell-cell signaling, cell division, hormone activity, inflammatory response, innate immune response and oxidoreductase activity. These results from our data-driven analysis are consistent with the hypothesis of EAOC pathogenesis proposed by the published studies, including genomic aberrations, immune and inflammation response, estrogen and oxidative stress, which are supposed to be related to the pathogenesis of EAOC pathogenesis [18,19,20].

There are several known genetic or genomic aberrations related to EAOC, including PTEN and KRAS. Loss of PTEN is found in 40% of cases of CCC and will lead to a deregulated PI3K-AKT pathway, one of the most significant deregulated functions in our study [21]. It has been demonstrated to play an important role in CCC and EC carcinogenesis and is responsible for 40% of ovarian carcinomas [21]. Our function-based study detects the deregulated PI3K-AKT signaling pathway (the third and 11th deregulated GO terms) instead of showing the aberration of the PTEN gene. In fact, intimate interactions exist among PTEN, PI3K and KRAS. PI3K is the major downstream effector of receptor tyrosine kinases (RTK, GO:0030971), a child of GO term “protein tyrosine kinase binding” (GO:1990782, the 14th and 19th deregulated GO terms for CCC and EC). If PI3K is activated, apoptosis will be inhibited and leads to cell proliferation or carcinogenesis [22]. KRAS is a GTPase that can turn on the downstream effectors such as PI3K by binding to GTP activated by GTPase activating proteins (GAPs) or turned off by conversion of GTP to GDP initiated by guanine nucleotide exchange factors (GEFs) [23]. Our results reveal the deregulated GTPase regulators (“Rho guanyl nucleotide exchange factor activity”, the first and 12th deregulated GO terms for CCC and EC) involved in the pathogenesis of EAOC instead of the KRAS gene aberration.

ES is an estrogen-dependent inflammatory disease. Aromatase highly expressed in endometriosis produces excessive estrogen and results in cell proliferation through stimulation of cytokine production [24]. These cytokines can stimulate the secretion of estrogen through aromatase in a positive feedback loop and establish an environment of hyperestrogenism contributing to abnormal cell proliferation [25]. Our study demonstrates the “response to steroid hormone stimulus” (255th deregulated GO terms) was deregulated in ES. Noticeably, the regulation of steroid hormone functions gets worse and moves upward in rankings in the functionomes from ES to CCC or EC (as the sixth and 22th GO term “steroid hormone receptor binding” in CCC and EC). The ranking analysis also reveals the deregulated hormone functions as the key elements involved in EACO pathogenesis. The inflammation process in endometriosis also induces macrophages to produce a variety of cytokines contributing to tumorigenesis and progression [26] and to produce cytotoxic reactive oxygen species (ROS), leading to DNA damage by oxidation of nucleotides [27]. Our study utilizing the EFA shows results consistent with this hypothesis: the hormone activity, inflammatory/immune response and oxidative stress are the primarily deregulated functions coexisting in ES, CCC and EC and progressively deregulated from ES to CCC and EC.

The deregulated GO term “inflammatory response” (GO:0006954) coexists in ES, CCC and EC. Especially, the GSR model detects two known inflammation-related pathways: MAPK and NF-kB in ES. Our study shows “MAP kinase kinase kinase kinase activity” (GO:0008349), a child GO term of MAPK (GO:0043405) pathway, as the 10th deregulated GO terms in ES. The MAPK pathway is responsible for transducing stimuli, such as proinflammatory cytokines to regulate cell functions including cell proliferation, survival and apoptosis [28]. NF-kappaB binding (GO:0051059) is the 64th deregulated function in ES. The immune microenvironment is critical for the carcinogenesis of EAOC. The cell proliferation resulting from aberrations humoral immunity and complement pathway activation is postulated to play an important role in the pathogenesis of EAOC [29]. Our study reveals consistent findings: the “activation of immune response” is the 14th deregulated GO term in ES; the “humoral immune response” is the 25th and 20th deregulated GO terms for CCC and EC.

**Figure 9:**
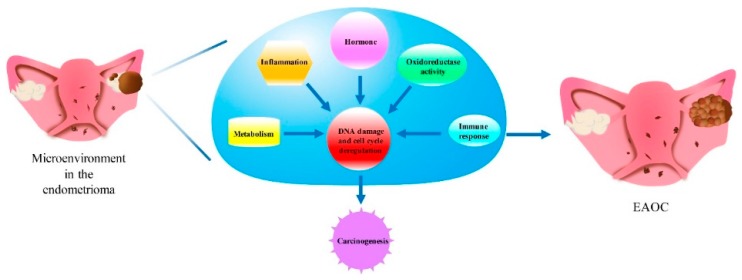
Possible contribution of the microenvironment in endometriosis to the development of EAOC. Endometriosis is an inflammatory condition arising from ectopic implantation of endometrial glands and stroma outside the uterine endometrium [29]. This microenvironment, especially the high concentration of free iron, which is derived from old menstrual blood accumulated in endometriosis (endometrioma), causes oxidative stress (ROS) and DNA damage [31,32]. Accumulation of DNA damage and inflammation/immune cytokines activated oncogenes [33,34]. Progressively reprogrammed metabolism and hormone changes over the years [35,36,37] eventually lead to the carcinogenesis of EAOC.

The 29th GO term “heme biosynthesis process” and the 35th GO term “heme metabolic process” indicated the deregulation of heme metabolism in ES. In the endometriotic lesion, the free heme and iron released from hemoglobin, as well as the autoxidation and Fenton reaction [30] of hemoglobin result in the production of excessive ROS, deregulation of redox homeostasis and DNA damage. In addition, oxidative stress also triggers antioxidant defense. Antioxidants prevent cell death by scavenging ROS; however, this also leads to abnormal cell proliferation and malignant transformation of ES to EAOC [31]. Oxidative stress was more predominant in CCC and EC. The most significant deregulated GO term related to oxidative stress in ES was the 200th GO term “oxidoreductase activity” (GO:0016491); the oxidative stress-related GO terms moved upward in rankings progressively from ES to CCC and EC, such as the 16th GO term “oxidoreductase activity acting on the CH-NH group of donors” (GO:0016645) in CCC and the 15th deregulated GO term “oxidoreductase activity acting on the aldehyde or OXO group of donors” (GO:0016903) in EC; this indicated that the role of oxidative stress was more and more important in the development of EAOC from ES to CCC or EC. These crucial deregulated functions, including inflammation, immune response, hormone activity, cell cycle control and oxidoreductase activity, interact with each other and act in a network contributing to the carcinogenesis of EAOC, as Figure 9 showed.

It has to be noted that the GSR model has its limitations. First, the GO terms’ gene set databases did not cover all human functions. For example, the GO term “receptor tyrosine kinase binding” (GO:0030971) was shown to be involved in the pathogenesis of EAOC, but it is not defined in the GO gene set database; only the parent GO term “protein tyrosine kinase binding” (GO:1990782) was defined and detected in our study. The second limitation is the detectability of this model. The GSR model converts gene expression levels to gene expression orderings, and this conversion will inevitably lead to a certain degree of information loss.

In conclusion, we established a bioinformatic platform of function-based, data-driven analysis of the molecular functionome to dissect the molecular pathogenetic pathways of EAOC. We demonstrated that the inflammatory/immune response, oxidative stress and hormone activity play an interactive role in modulating the malignant transformation and cancer progression in EAOC. Our results support the postulation that endometriosis shares similar molecular signatures with EAOC, which was validated by data-driven analysis. Our data also raised the possibility of using the inflammatory/immune response, oxidative stress and hormone activity as molecular biomarkers in monitoring the malignant transformation of endometriosis. Further immunohistochemical staining and functional validation are warranted to support the significance of identified functionomes clinically.

## 4. Materials and Methods

### 4.1. Computing the GSR Indices

The detail of the GSR model and the computing procedures are described in our previous study [13,14]. Briefly, the GSR model converted gene expression profiles to quantified functions with the differential rank conservation (DIRAC) [38] algorithm, which measures the ordering change of the gene elements in a gene set between the gene expression orderings in ES, CCC or EC and the most common gene expression ordering in the normal control population. Microarray gene expression profiles were downloaded from the GEO database in .SOFT format, and then, the gene expression levels were extracted according to the corresponding gene elements in the GO term gene set and converted to the ordinal data based on their expression levels. The GSR index is the ratio of gene expression ordering in a gene set between each case or normal control sample and the most common gene expression ordering among the normal tissue samples. Establishment of the baseline gene set expression ordering templates and measurement of GSR indices were executed in the R environment.

### 4.2. Microarray Datasets, Gene Set Definition and Data Processing

The selection criteria for downloaded microarray gene expression datasets were: (1) both the case and normal control samples should originate from identical tissue, i.e., ovarian tissue for CCC and EC or endometrium for ES; (2) the datasets should provide definite information on the diagnosis for each sample; (3) since this study utilized the common genes among the selected datasets, a dataset was discarded if it resulted in the number of common genes being less than 8000 when it was integrated; and (4) a gene expression dataset was discarded if it contained missing data.

### 4.3. Statistical Analysis

The differences of the GSR indices between the three diseases and the control groups were tested by the Mann–Whitney *U*-test and corrected by multiple hypotheses using the false discovery rate (Benjamini–Hochberg procedure). The significance level was set at <0.01.

### 4.4. Classification and Prediction by Machine Learning

GSR indices computed through the GO term gene sets were classified and predicted by support vector machine (SVM) with kernlab (Alexandros K, 2004), an R package for kernel-based machine learning methods, and was used to classify patterns of the GSR indices with the setting of kernel = “vanilladot” (linear kernel function). The performance of classification and prediction by SVM was measured by five-fold cross-validation: samples were randomly sampled and divided into five parts; four parts were used for training sets and the remainder for prediction. The performance of binary classification was assessed with sensitivity, specificity, accuracy and AUC. Sensitivity, specificity, accuracy and AUC were computed using the cumulative results of repeating 10 classifications. AUC was computed by an R package pROC [39] (Robin et al., 2011). The accuracy of multiclass classification was computed from the fraction of correct predictions within the total prediction number.

### 4.5. Hierarchical Clustering, Dendrogram and Heatmaps

The GSR indices in each gene set were averaged then underwent hierarchical clustering with the function “heatmap.2” in R package “gplots” (Version 2.17.0) as the default. This function executed hierarchical clustering and drew dendrograms and heatmaps.

All possible logical relations among the deregulated functions of the three diseases were evaluated and displayed by Venn diagram using the R package “VennDiagram” (Version 1.6.16).

### 4.6. Exploratory Factor Analysis and Reconstruction of GO Trees

The deregulated GO terms of *p* < 0.01 were selected for EFA to uncover the underlying structure of pathogenesis for ES, CCC and EC. The EFA was executed with the R package “psych” (Version 1.5.8). The number of factors to be extracted was determined by the function “pa.parellel”. The factoring method used in this study was set to “pa”, and the correlation matrix rotation method was “promax”. All of the factor elements for each disease were merged together to reconstruct the GO tree by “RamiGO” [40], an R package providing functions to interact with the AmiGO 2 web server [41] and retrieve GO trees.

### 4.7. Ranking Analysis

The progressive deregulated functions were selected by tracing their rankings in the functionome during progression from ES to EAOC. To compare the rankings of different diseases, we selected the GO terms with the following criteria: (1) *p* < 0.01; (2) the difference of ranks between CCC and EC was less than 100; (3) the average of ranks for CCC and EC was less than 300; and (4) the difference of ranks between ES and CCC or EC was more than 0. The ranks of selected GO terms were displayed on a line chart to show the paths of rank changing from ES to CCC or EC.

### 4.8. Reconstruction of the Interaction Network

The network was established by computing the mutual information based on entropy estimates from k-nearest neighbor distances, and the interaction network (multiplicative model) was constructed by the algorithm for the reconstruction of accurate cellular networks (ARACNE) using the R package “parmigene” (Version 1.0.2). The network was output in the graph modeling language (GML) format and displayed using Cytoscape (Version 3.3.0).

## 5. Conclusions

Complex diseases like ES, CCC, EC or EAOC usually involve a spectrum of variably-deregulated functions. Thus, we investigated the pathogenesis of EAOC with the functions consisting of 1454 GO term gene sets. We demonstrated that the informativeness of the GSR indices was sufficient for accurate recognition of complex disease patterns. Using a series of analytic procedures and filters, this data-driven analysis demonstrated genome-wide evidence in support of the proposed pathways or dysfunctions involved in EAOC. These results demonstrated the deregulated metabolism, cell cycle control, cell-cell signaling, hormone activity, inflammatory response, immune response and oxidoreductase activity as being the principle members of EAOC pathogenesis.

## Figures and Tables

**Table 1:** Sample number and mean of the gene set regularity indices for each group. SD: standard deviation.

Gene Set	Disease	Case	Control	Case Mean (SD)	Control Mean (SD)	*p*-Value
GO term	ES	80	100	0.7815 (0.0970)	0.8898 (0.0740)	<0.01
	CCC	80	100	0.7438 (0.1177)	0.7759 (0.1315)	<0.01
	EC	80	100	0.7433 (0.1256)	0.7764 (0.1312)	<0.01

**Table 2:** The 15 most deregulated Gene Ontology terms for the three diseases ranked by *p*-values.

	Endometriosis	Clear Cell Carcinoma	Endometrioid Carcinoma
1	Symporter activity	Rho guanyl nucleotide exchange factor activity	Cofactor transporter activity
2	Anion cation symporter activity	Cofactor transport	Carbohydrate biosynthetic process
3	Secondary active transmembrane transporter activity	Inositol or phosphatidylinositol phosphatase activity	Regulation of viral reproduction
4	Hydrogen ion transmembrane transporter activity	Regulation of viral reproduction	Secretin like receptor activity
5	Golgi stack	Small conjugating protein binding	Coenzyme binding
6	Monovalent inorganic cation transmembrane transporter activity	Steroid hormone receptor binding	Cofactor binding
7	Phosphate transmembrane transporter activity	Ubiquitin binding	Sulfotransferase activity
8	Cytochrome c oxidase activity	Vitamin transport	Calcium channel activity
9	Late endosome	Histone deacetylase binding	Calcium-independent cell-cell adhesion
10	MAP kinase kinase kinase activity	Protein tyrosine kinase activity	Transferase activity transferring sulfur containing groups
11	Monocarboxylic acid transmembrane transporter activity	Negative regulation of cellular component organization and biogenesis	Inositol or phosphatidylinositol phosphatase activity
12	Protein amino acid autophosphorylation	Insoluble fraction	Rho guanyl nucleotide exchange factor activity
13	Protein autoprocessing	Carbohydrate biosynthetic process	Cofactor transport
14	Activation of immune response	Transmembrane receptor protein tyrosine kinase activity	Oxidoreductase activity acting on the aldehyde or OXO group of donors
15	Carboxylic acid transmembrane transporter activity	SH3 SH2 adaptor activity	Vitamin transport

**Table 3:** Accuracies of the binary and multiclass classification and prediction by machine learning.

Gene Set	Classification	Group	Sensitivity (SD)	Specificity (SD)	Accuracy (SD)	AUC
**GO term**	**Binary**	**ES**	0.9841(0.0255)	0.9947(0.166)	0.9888(0.0143)	0.9881
		**CCC**	0.9933(0.0210)	1.0000(0.0000)	0.9972(0.0008)	0.9965
		**EC**	0.9663(0.0612)	0.9954(0.0143)	0.9861(0.0236)	0.9834
	**Multiclass**	**ES-CCC-EC-control**	NA	NA	0.9820(0.0005)	NA

## Supplementary Materials

Supplementary materials can be found at www.mdpi.com/1422-0067/18/11/2345/s1.

## Abbreviations

AUC	area under the curve
CCC	clear cell carcinoma
EAOC	endometriosis-associated ovarian carcinoma
EC	endometrioid carcinoma
EFA	exploratory factor analysis
EOC	epithelial ovarian carcinomas
ES	endometriosis
FIGO	Federation of Gynecology and Obstetrics
GEO	gene expression omnibus
GO	gene ontology
GSR	gene set regularity
GTPase	guanosine triphosphatase
MAPK	mitogen-activated protein kinase
MSigDB	molecular signatures database
NCBI	National Center for Biotechnology Information
SC	serous carcinoma
SD	standard deviation
SVM	support vector machine
