# Supplementary figures and images for: Discovering the Deregulated Molecular Functions Involved in Malignant Transformation of Endometriosis to Endometriosis-Associated Ovarian Carcinoma Using a Data-Driven, Function-Based Analysis
Source: Int J Mol Sci. 2017 Nov 6;18(11):2345. doi: 10.3390/ijms18112345 (PMC5713314; doi:10.3390/ijms18112345)

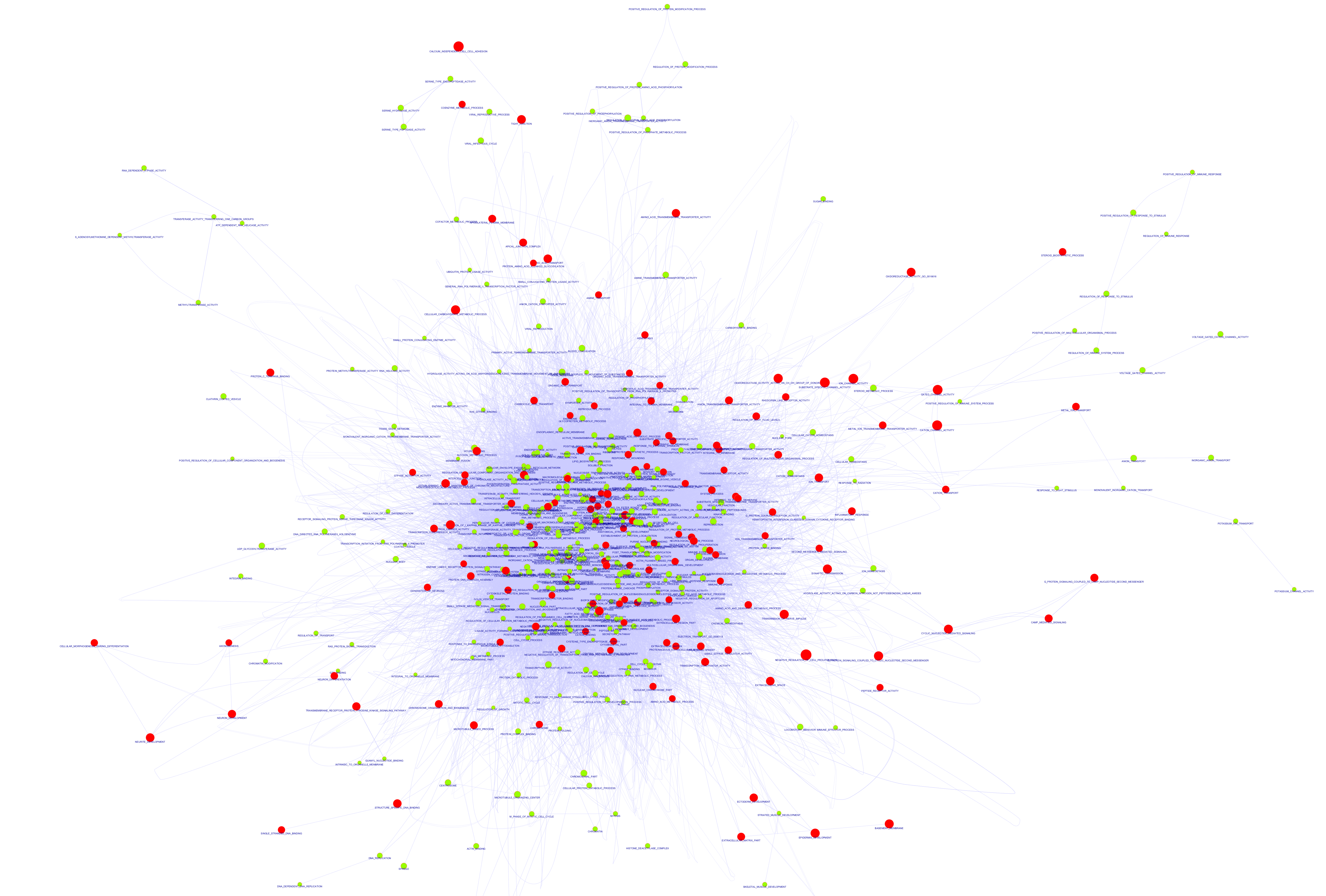

Supplement: Supplementary file 1 [file ijms-18-02345-s001.zip › Figure S4.tif]
